# Association between Internet Gaming Disorder or Pathological Video-Game Use and Comorbid Psychopathology: A Comprehensive Review

**DOI:** 10.3390/ijerph15040668

**Published:** 2018-04-03

**Authors:** Vega González-Bueso, Juan José Santamaría, Daniel Fernández, Laura Merino, Elena Montero, Joan Ribas

**Affiliations:** 1 Atención e Investigación en Socioadicciones (AIS), Mental Health and Addictions Network, Generalitat de Catalunya (XHUB), C/Forn-7-9 Local, 08014 Barcelona, Spain; vgonzalez@ais-info.org (V.G.-B.); lmerino@ais-info.org (L.M.); emontero@ais-info.org (E.M.); 38039jrs@comb.cat (J.R.); 2 Research and Development Unit, Parc Sanitari Sant Joan de Déu, Fundació Sant Joan de Déu, CIBERSAM, Dr. Antoni Pujadas, 42, Sant Boi de Llobregat, 08830 Barcelona, Spain; df.martinez@pssjd.org; 3 School of Mathematics and Statistics, Victoria University of Wellington, Wellington 6140, New Zealand

**Keywords:** pathological video-game use, Internet Gaming Disorder, comorbid psychopathology, review

## Abstract

The addictive use of video games is recognized as a problem with clinical relevance and is included in international diagnostic manuals and classifications of diseases. The association between “Internet addiction” and mental health has been well documented across a range of investigations. However, a major drawback of these studies is that no controls have been placed on the type of Internet use investigated. The aim of this study is to review systematically the current literature in order to explore the association between Internet Gaming Disorder (IGD) and psychopathology. An electronic literature search was conducted using PubMed, PsychINFO, ScienceDirect, Web of Science and Google Scholar (r.n. CRD42018082398). The effect sizes for the observed correlations were identified or computed. Twenty-four articles met the eligibility criteria. The studies included comprised 21 cross-sectional and three prospective designs. Most of the research was conducted in Europe. The significant correlations reported comprised: 92% between IGD and anxiety, 89% with depression, 85% with symptoms of attention deficit hyperactivity disorder (ADHD), and 75% with social phobia/anxiety and obsessive-compulsive symptoms. Most of the studies reported higher rates of IGD in males. The lack of longitudinal studies and the contradictory results obtained prevent detection of the directionality of the associations and, furthermore, show the complex relationship between both phenomena.

## 1. Introduction

The problematic use of video games is recognized by mental health professionals as an addictive behavior with clinical relevance. This is due to the negative consequences it may have for affected people in several functional areas such as relationship conflicts, sleep problems or occupational functioning [1,2]. However, in the current literature, the terms “Internet addiction” (IA) and “pathological Internet use” (PIU) have commonly been used to refer to all sorts of activities including, but not limited to, the use of video games. All these activities are derived from the excessive use of devices connected to the Internet (i.e., computers, smartphones and other devices to play on and navigate). This classification has frequently been criticized as being too broad and not distinguishing between problematic activities and the medium itself on which they take place [3,4], despite the fact that persons engaged in these activities have different sociodemographic characteristics and motivations [5]. For example, the Internet preference activities for males are those related to entertainment and leisure, whereas women tend to choose activities related to interpersonal communication and educational assistance; additionally, these differences may be mediated by age [6].

The non-inclusion of IA as a diagnosis, and the inclusion of “Internet Video-Game Disorder” (Internet Gaming Disorder, IGD) in Section III of the diagnostic manual DSM-5 [7] as a condition that requires further study, seems to support considering both disorders as different problems. Likewise, the most recent inclusion of Gaming Disorder in the beta version of the ICD-11 (International Classification of Diseases) of the World Health Organization [8] seems to confirm this trend. In this document, the problem is defined as “a pattern of persistent or recurrent gaming behavior (‘digital gaming’ or ‘video-gaming’), which may be online (i.e., over the Internet) or offline, manifested by: (1) impaired control over gaming (e.g., onset, frequency, intensity, duration, termination, context); (2) increasing priority given to gaming to the extent that gaming takes precedence over other life interests and daily activities; and (3) continuation or escalation of gaming despite the occurrence of negative consequences. The behavior pattern is of sufficient severity to result in significant impairment in personal, family, social, educational, occupational or other important areas of functioning. The pattern of gaming behavior may be either continuous or, on the other hand, episodic and recurrent. The gaming behavior and other features are normally evident over a period of at least 12 months for a diagnosis to be assigned, although the required duration may be shortened if all diagnostic requirements are met and symptoms are severe”.

The psychopathology associated with addictive behaviors, with or without substance, can result from a problem or, alternatively, lead to further issues [9,10]. If the association between two disorders is higher than expected by chance, it is likely that there are mechanisms contributing to that association. Four general models of increased comorbidity have been described [11,12,13]: common factor models, secondary substance-use disorder models, secondary psychiatric disorder models, and bidirectional models. In the first instance, both disorders share risk factors and the higher comorbidity is the result. In the second case, the addictive disorder contributes to other psychiatric disorders. In the third condition, the psychiatric disorder precipitates the addictive behavior. Finally, either disorder can increase vulnerability to the other disorder; in such cases the higher comorbidity reported may be due to inappropriate sampling, assessment, study design or other biases in the published studies.

In the case of behavioral addictions, the temporal linearity of that relationship remains unclear. Associations between IA or PIU and various psychiatric symptoms have been reported in the literature. Specifically, they have been related to depression, attention deficit hyperactivity disorder (ADHD), anxiety, obsessive-compulsive symptoms, and hostility or aggression [14]. Depression seems to be the most common comorbidity in all age groups (adolescents, adults and the general population). However, the designs used to explore these relationships are not sufficiently comprehensive or complex to confirm the hypothesis for the above models. It is possible that a specific psychiatric problem might have an influence on developing an IA, or that a person with an IA diagnosis, due to various negative consequences, will later develop a comorbid psychiatric disorder. It is also possible that both problems share biological, sociodemographic or psychological underlying mechanisms that make people vulnerable to both pathologies; these may thus become evident at the same time [15]. A major drawback of these studies is that, in most, the type of Internet use is not controlled or, alternatively, the results are not separated by use. In many studies, playing video games is the most common activity among people with IA [16,17,18,19]; still, the results have been analyzed without taking this aspect into account.

Therefore, some interesting questions remain. One is whether IGD has similar comorbidities to IA or, rather, the comorbidities are different. In the latter case, one may wonder if other Internet-based issues are affecting in some way the results of studies focused on IA in general. An additional question pertains to the directionality of both conditions (IGD and psychopathology).

The aim of this study is to review systematically the current literature to elicit epidemiological evidence supporting or refuting the association between Internet gaming addiction and psychopathology. An additional objective is to explore the relationship between these conditions. Such results can furnish clinicians with updated information and provide a direction for future investigative endeavors.

## 2. Materials and Methods

This systematic review was conducted in accordance with the Preferred Reporting Items for Systematic Reviews and Meta-Analyses-P 2015 statement for systematic review and meta-analysis protocols [20]. The databases reviewed between October and December 2017 were PubMed, PsychINFO, ScienceDirect, Web of Science, and Google Scholar, using the following search terms and logic: “(Internet OR online) gaming addiction AND (psychopathology OR comorbidity)”. Without considering the results in Google Scholar, these database search parameters yielded a total of 688 results, including the following results in each database: PubMed (54 results), PsychINFO (354 results), and ScienceDirect (280 results). Due to the large number of results provided by Google Scholar (more than 17,500 results), we reviewed only the first 30 pages of results. Additional articles were identified through searching the citations in the literature selected.

The studies were systematically and independently reviewed by the authors (Vega González-Bueso and Juan José Santamaría); paying attention to the study type, study population, methodology, outcome measures, effect sizes and interpretation of results. In cases of discrepancies, these were resolved through consensus or referral to a third reviewer (Laura Merino). The inclusion criteria were: (i) the inclusion of empirically collected data; (ii) IGD assessed by standardized questionnaires or other proposed criteria based on international disease classifications; (iii) psychiatric comorbidity assessed by standardized questionnaires; (iv) availability of the full text; (v) published after the year 2010 (this allowed us to review the most recent research in a field where the subject of addiction evolves rapidly); (vi) written in English or in Spanish (the two languages known by the authors); and (vii) article published in a peer-reviewed journal.

Studies were also included if the object of research was IA, only if it was specified that the Internet was used to play video games, and/or the results were separated according to Internet use and whether video games were one of those activities.

The exclusion criteria were: (i) articles containing only anecdotal evidence on psychopathology associated with IGD; (ii) authors not providing a specific definition or criteria for IGD; (iii) case reports and case series; (iv) studies only reporting results on phenomenons such as motivation to play video games, decision-making, stress, lifestyle, impulsivity and sexual attitude, without reporting other psychiatric comorbidity.

A review protocol exists at the PROSPERO International prospective register of systematic reviews [21] registration number CRD42018082398.

**Figure 1:**
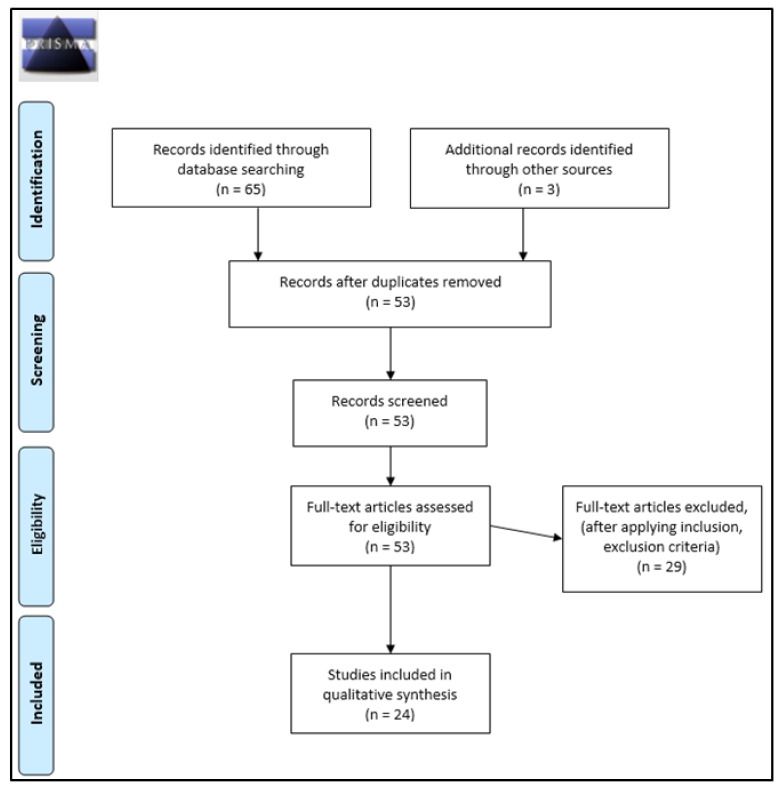
PRISMA 2009 protocols flow diagram.

In order to facilitate the comparisons with pathological Internet use, the reviewing method applied by Carli et al. in 2013 [14] was followed: the effect sizes of the associations between IGD and psychopathology were identified by the reviewed publications or calculated using the data provided by the authors, when available. In order to compare the different associations, the effect sizes d and R^2^ were stated as small, moderate, or large, according to Cohen [22]; OR were converted into these groups according to Chinn [23]. The effect sizes were interpreted accordingly: small (d = 0.2, R^2^ = 0.01, OR = 1.45), moderate (d = 0.5, R^2^ = 0.06, OR = 2.50), and large (d = 0.8, R^2^ = 0.14, OR = 4.25). Full association was considered when a correlation was found for both genders after multivariate analyses. If a correlation was identified for only one gender, it was classified as a partial association. The geographical distribution of studies was also mapped (Figure 1).

## 3. Results

After deleting duplicate studies, a total of 68 articles were screened and identified through the present systematic search. After applying inclusion and exclusion criteria, a total of 24 studies were selected and included. Table 1 shows a summary of the main characteristics of the studies examining the relationship between IGD and comorbid psychopathology, including effect sizes.

### 3.1. Design of the Included Studies

Nineteen of the 24 articles included were cross-sectional studies [24,25,26,27,28,29,30,31,32,33,34,35,36,37,38,39,40,41,42], the rest were two longitudinal studies [43,44], two case-control studies [45,46], and a cohort study [47]. The research was performed, in descending order, in South Korea (4), Australia (3), Germany (3), Norway (2), Taiwan (2), Canada (1), USA (1), Singapore (1), Spain (1), United Kingdom (1), France (1), Finland (1), Deutschland (1) Austria (1) and Sweden (1). Most of the studies were performed in European countries (12).

### 3.2. Characteristics of the Used Samples

The 24 studies had a total of 53,889 participants. All studies examined both genders. The number of participants in each study ranged from 102 to 12,938 (M = 2155.56; standard deviation (SD): 3176.05). Nine of the studies in this review [24,28,29,33,37,38,40,43,47] targeted adolescent groups, six studies [26,27,30,34,42,46] targeted adults, one [44] targeted children and eight studies [25,31,32,35,36,39,41,45] were carried out in the general population. A total of three studies were conducted in clinical populations, using people in outpatient treatment for IGD [45] or other mental health problems, namely Gambling Disorder [26] and other unspecified psychiatric problems [38].

### 3.3. Methods of Assessing Internet Gaming Disorder (IGD)

Since 2013, the DSM-5 includes a proposal of diagnostic criteria for IGD. However, only five out of 15 of the reviewed articles published after this year used these criteria [27,29,34,39,46]; three use psychometric questionnaires based on them [30,38,40] to assess the problem.

These diagnostic criteria pertain to repetitive use of Internet-based games, often with other players, that leads to significant issues with functioning. Five of the following criteria must be met within one year: “(i) Preoccupation or obsession with Internet games. (ii) Withdrawal symptoms when not playing Internet games. (iii) A build-up of tolerance (i.e., more time needs to be spent playing the games). (iv) The person has tried to stop or curb playing Internet games but has failed to do so. (v) The person has had a loss of interest in other life activities, such as hobbies. (vi) A person has had continued overuse of Internet games even with awareness of how much they impact a person’s life. (vii) The person has lied to others about his or her Internet game usage. (viii) The person uses Internet games to relieve anxiety or guilt (i.e., it is a way to escape). (ix) The person has lost or put at risk opportunities or relationships because of Internet games”.

The questionnaires based on these criteria were the Internet Gaming Disorder Test-10 (IGDT-10) [48]; the Gaming Addiction Identification (GAIT) [49] and the Internet Gaming Disorder Scale (IGDS) [50].

The IGDT-10 includes the nine diagnostic criteria of the DSM-5. Each criterion was operationalized using a single item, except for the last criterion referring to “jeopardy or losing a significant relationship, job, or educational or career opportunity because of participation in Internet games.” This criterion was operationalized with two items, given its complexity and description of more than one construct.

The GAIT is a screening instrument used to identify addictive factors related to gaming addiction in adolescents. Primarily developed based on items from the AUDIT Alcohol Consumption Questions (AUDIT-C) [51], and the criteria for gambling disorder suggested by the DSM-5, GAIT covers seven of the nine criteria in the proposed IGD criteria. These items are: preoccupation, withdrawal, tolerance, unsuccessful attempts to control the behavior, loss of interests, harm, and loss of a significant relationship or educational opportunity due to gaming. Questions regarding lying/deception to hide the gaming, and escape/mood modification, are not included.

Finally, the IGDS measures each of the nine DSM-5 definitions with three items, either through separating core aspects of a criterion into different items or by applying changes in phrasing or synonyms. Furthermore, the proposed terms “Internet gaming” or “Internet games” were replaced with “gaming” or “games.”

The remaining studies employed either measures based on the DSM-IV Gambling Disorder criteria (Pathological Technology Use (PTU), Gaming Addiction Scale (GAS)) or based on DSM-IV Addiction criteria (Gaming Addiction Scale for Adolescents (GASA), Video-game Dependency Test (VDT), Assessment of Internet and Computer Game Addiction (AICGA), Video-Game Use Questionnaire (VGUQ)), or questionnaires used to measure IA problems (Computer/Gaming-station Addiction Scale (CGAS), Generalized Problematic Internet Use Scale (GPIUS), Young Internet Addiction Scale (YIAS), Compulsive Internet Use Scale (CIUS), Problematic Internet use scale (ISS-20), Young Diagnostic Questionnaire (YDQ), Chen’s Internet Addiction Scale (CIAS), and Problem Video-Game Playing Test (PVGT)).

### 3.4. Methods Assessing Psychopathology

Different psychometric assessments were used in the reviewed articles to measure psychopathology.

Depression was measured using various assessment tools, i.e., the Hopkins Symptom Checklist [52], the Asian Adolescent Depression Scale [53], the Beck Depressive Inventory [54], the Beck Depressive Inventory-II [55], the Center for Epidemiologic Studies-Depression Scale-10 [56], the Depressive Mood List [57], the Questionnaire for Depression Diagnostics [58], the Depression Self-Rating Scale [59], the Patient Health Questionnaire-9 [60] and the Depression and Somatic Symptoms Scale [61].

To assess anxiety, in each study, different measures were used, these are the State-Trait Anxiety Inventory [62], the Screen for Child Anxiety-Related Emotional Disorders [63], the Beck Anxiety Scale [64], the Spence Children’s Anxiety Scale [65], and the Generalized Anxiety Disorder Scale-7 [66]. In addition, some authors used questionnaires evaluating both depression and anxiety, the Revised Children’s Anxiety and Depression Scale [67], the School Health Promotion [68], the Hospital Anxiety and Depression Scale [69], the Youth Self-Report [70] and the Reynolds Adolescent Adjustment Screening Inventory [71].

To measure ADHD symptoms or hyperactivity, three authors [38,42,43] used the ADHD Self-Report Scale [72], two authors [24,37] used the Strengths and Difficulties Questionnaire [73], one author [45] used the Dupaul’s ADHD scale [74], and one author [46] used the ADHD DSM-IV-TR criteria diagnosis for adult and childhood [75].

To assess social phobia and social anxiety, two studies [41,43] used the Social Phobia Inventory [76], one study [25] used the Social Phobia Scale [77], and one study [44] used the Revised Social Anxiety Scale for Children [78].

Several studies used questionnaires to assess multiple conditions: in three articles [26,34,35] the Symptom Checklist 90-Revision [79] was employed to assess several conditions (somatization, obsessive-compulsive disorder, interpersonal sensitivity, depression, anxiety, hostility, phobic anxiety, paranoid ideation, and psychoticism), and one study [27] used the Brief Symptoms Inventory [80] to measure the same psychopathologies. Another study [28] evaluated depression, anxiety and obsessive-compulsive disorder through the Revised Children’s Anxiety and Depression Scale [67]. Finally, one article [24] assessed emotional problems and hyperactivity using the Strengths and Difficulties Questionnaire [73].

Finally, in one study [38] the association between IGD and psychoticism was explored through the Psychotic-like Experiences Test [81].

### 3.5. Effect Size of the Associations of Psychopathology with IGD

Regarding the associations between the analyzed mental disorders and IGD, the effect sizes reported in the reviewed papers comprised different levels of association: 35 large [24,25,26,27,30,31,41,42,43,45,46,47,82], 13 moderate [26,29,31,37,45], eight small [25,33,38,40], and seven non-association [36,38,43,44,83]. In order to summarize these results, Table 2 shows the observed associations identified between IGD and psychopathology only for the main four outcomes. The largest correlations were identified between IGD and anxiety and depression and ADHD, whereas the weakest were observed between IGD and obsessive-compulsive disorder.

### 3.6. Psychopathology, IGD and Sample Characteristics (Age, Gender)

Twenty-one studies were conducted in healthy populations; only three analyzed clinical populations (IGD or other mental health problems).

Regarding age, the analyzed studies included in the present review focused on three age groups as target populations: general population, adolescents and adults. 

Eight articles examined groups of general population formed by children, adolescents and adults together, exploring the association between IGD and 1 depression and anxiety [31,32,39,45], depression [36,41], anxiety [25], social phobia [25,41], ADHD [45] and several psychiatric symptoms using the SCL-90-R [35]. One of these studies focused on a clinical sample of IGD patients [45]. All studies found a large effect size in the correlation between IGD in the general population and depression, except for one that found a non-correlation between both disorders. Large correlation effects with IGD in the general population were also found with ADHD and social phobia. Two studies analyzing anxiety found large effect sizes and two found moderate effect sizes. Large effect sizes were also found with the remaining SCL-90-R scales.

Six studies were focused on adults, analyzing the association between IGD and depression and anxiety [34], depression [30], ADHD [42,46] and several psychiatric symptoms [26,27]; here the SCL-90-R and the Brief Symptom Inventory (BSI) questionnaires were used. One of these studies focused on a clinical sample of pathological gamblers [26]. The authors identified correlations between IGD and depression and anxiety with large and moderate effect sizes, large effect sizes with ADHD, paranoid ideation and obsessive-compulsive symptoms, and finally, large and moderate effect sizes with the remaining SCL-90-R scales.

Adolescent participant groups were used in the remaining 10 studies. One of these studies [38] focused on adolescents with unspecified psychiatric problems. An association between depression and IGD in adolescents was found in seven articles and non-association in one; the effect sizes varied between large (2), moderate (2) and small (2) and no association (1). Anxiety correlated with IGD in adolescents in four of the five studies exploring this relationship; the sizes of the effects varied between large (1), moderate (1) and small (2). The association with ADHD was found in four out of five studies, with effect sizes: large (1), moderate (1) and small (2). Social phobia or social anxiety showed a large association and no association in two studies. Finally, non-association was found with obsessive-compulsive disorder (OCD) and psychoticism in the adolescent population.

With respect to gender, all studies reported higher video-game use among males. Seventeen studies [25,26,27,28,30,32,33,34,35,37,38,39,40,42,43,45,46] found higher rates of IGD among males. Two [24,29] reported no gender differences. The association between psychopathology and IGD was found for both sexes in all the articles (full association), except one [35] that only analyzed the relationship between males.

### 3.7. IGD and Depression

Nineteen of the 21 studies examined some form of depression as a comorbid symptom. Thirteen studies found a full association [26,27,29,30,31,37,38,40,41,43,45,47,82], and two [28,36] found no association. Specifically, King et al. [28] reported association with depression in PIU groups, demonstrating significantly more severe depression and anxiety symptoms than either the non-problematic user’s group or the pathological video gamers group. In contrast, the pathological video gamers group scores did not differ significantly from the non-problematic users group.

Four studies were not cross-sectional, there were two longitudinal studies [43,44], one cohort study [47], and one case-control [45]. The results of these studies showed large effect size associations with depression. In the case of the longitudinal studies, Gentile et al. [43] reported elevated depressive symptoms after the pathological video-gaming problems started and these symptoms persisted and increased only if the pathological abuse persisted, while Van Rooij et al. [44], in their longitudinal study exploring two different times (years 2008 and 2009) found correlations with depressive mood only in Time 2 when comparing addicted heavy gamers with non-addicted heavy gamers. In the cohort study, the authors reported a correlation between video game addiction and depression with a large effect size only in Time 1, but they did not find any significant correlation between these two variables two years later. Among the rest of correlations detected, the effect sizes for the association with depression comprised eight large [27,30,31,41,43,45,47,82], three moderate [26,29,37], and two small [38,40] observed effects.

### 3.8. IGD and Anxiety

Regarding the correlation between IGD and anxiety, 11 studies found a full association, one study found a partial association, and one study found no association. The studies finding full association were: a longitudinal study [43] identifying a large effect size; a case-control study [45] identifying a moderate effect size; a cross-sectional study [25], where the authors reported a large effect size in the correlation with the anxiety trait, but a small effect size with anxiety state; and eight cross-sectional studies [26,27,29,31,32,34,38,40] identifying large effect sizes (1), moderate effect sizes (3), and small effect sizes (2). Just as in the case of depression, in the longitudinal study carried out by Gentile et al. [43], the anxiety symptoms appeared after pathological video-gaming problems. A partial association only in males was found in a study [35] and here there was a moderate effect size. Finally, no association with anxiety was found in one cross-sectional study [28].

### 3.9. IGD and Attention Deficit Hyperactivity Disorder (ADHD)

The relationship between IGD and ADHD and hyperactivity symptoms were analyzed in eight studies. Seven of them reported full association, with four finding large [24,42,45,46], two finding small [38,40], and one reporting moderate, effect sizes [37]. The studies comprised two case-control, five cross-sectional and one longitudinal design; the latter found no association between the two variables [43].

### 3.10. IGD and Social Phobia and Social Anxiety

Four studies included social phobia or social anxiety as a comorbid symptom in their studies. These studies comprised two longitudinal [43,44] and three cross-sectional designs [25,41,44]. One longitudinal and two cross-sectional studies found full association with IGD, reporting large effect sizes. Furthermore, the longitudinal study, similar to the results found regarding anxiety and depression, found that social phobia symptoms worsen after a youth becomes a pathological gamer, and improve if an individual stops this activity. In the remaining longitudinal study, no association was found between social anxiety and IGD.

### 3.11. IGD and Obsessive-Compulsive Symptoms

Four studies examined obsessive-compulsive symptoms as a comorbid problem. Three studies [26,27,35] found a full association with large effect sizes, and one [28] found no association.

### 3.12. Publication Bias

**Figure 2:**
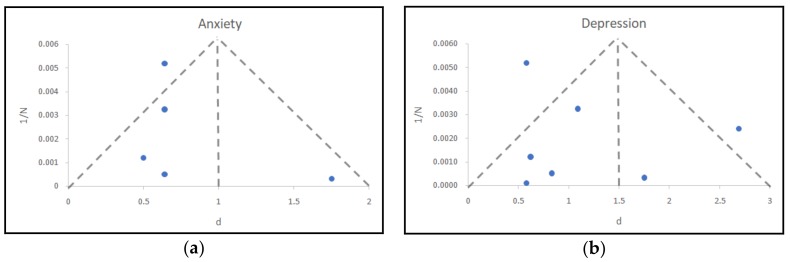
Funnel plots with pseudo-95% confidence limits: (**a**) anxiety, (**b**) depression.

In order to detect possible publication bias, a funnel plot was conducted for depression and anxiety, as there was only a sufficient number of studies reporting results for these two pathologies (according to Grading of Recommendations, Assessment, Development and Evaluation Working Group (GRADE guides), a minimum of five to 10 studies with the same statistic reported are needed). A total of seven studies analyzing the relationship between depression and IGD, and a total of five analyzing anxiety and IGD, reported d values or data to calculate them. Figure 2 depicts the distribution of the reported or calculated correlations for depression and anxiety. The x-axis and y-axis represent the reported d values and the inverse of the sample size, respectively.

The location of the studies shows a bias towards the left side of the funnel plot, i.e., low values of d, indicating a possible publication bias. Even so, we would like to remark that the number of studies is very small to conclude with definitive results in both psychopathologies [84,85], and thus this information must be interpreted cautiously.

## 4. Discussion

The main purpose of this review was to explore the state of current literature about the relationship between IGD and comorbid psychopathologies, as this knowledge is crucial to the positioning of the disorder as a behavioral addiction. A secondary aim was to analyze the effect size of these correlations and the potential effect of publication bias. In the reviewed papers on IGD and comorbid psychological pathologies, 92% of the studies describe significant correlations with anxiety, 89% with depression, 87% with ADHD or hyperactivity symptoms, and 75% with social phobia/anxiety and obsessive-compulsive symptoms. However, the potential publication bias detected in the preliminary analysis demands caution in interpretation of the results. Notwithstanding this, it should be noted that despite the inclusion of IGD in Section III of the Diagnostic and Statistical Manual DSM-5 [7] and in the beta version of the ICD-11 (International Classification of Diseases) [8], only a marginally small number of publications were centered on IGD in the literature, and several authors continue analyzing IA or PIU as a whole, without distinguishing the different possible problematic activities that users experience with this medium.

With regard to the main purpose, IGD showed strong correlations with most of the analyzed psychopathologies, in comparison with PIU, where the strongest association was found with depression [14]. The effect sizes examined indicated that the strongest associations were found with anxiety, depression, and ADHD or hyperactivity symptoms and social phobia/anxiety.

The fact as to whether the addictive behaviors (with or without substance use) may be a consequence or a trigger of psychopathology [15] cannot be unraveled yet. The lack of longitudinal studies analyzing the temporal linearity of these events in AI or PIU precludes clarifying whether a specific psychiatric problem helps to develop an AI or, alternatively, a person with a diagnosis of AI —due to negative consequences stemming from it—later developed a comorbid psychiatric disorder. A third possibility is that both problems share underlying biological, sociodemographic or psychological mechanisms that make people vulnerable to both pathologies (which manifest at the same time). In the case of this review, two longitudinal studies and one cohort study required data on whether IGD was the cause or consequence of psychopathological problems; as a result, contradictory results were obtained. On the one hand, the results of the longitudinal study performed by Gentile et al. [43] showed that the adolescents who became and stayed pathological gamers during the study period, in the last time measured, ended up with increased levels of depression, anxiety and social phobia, while those who were pathological at the start but stopped being pathological, ended up with reduced levels of depression, anxiety and social phobia. These results seem to demonstrate that gaming predicts other mental health disorders longitudinally, rather than simply being correlated with them. On the other hand, van Rooij et al. [44] found a relation between addicted heavy gamers and depression in the second year, but no correlation with social anxiety at any time. Finally, Brunborg et al. [47] only found a correlation between depression and IGD at Time 1, but not at other times.

These ambiguous results show the complex relationship between the intrinsic characteristics of online video games, the consequences of their abuse, and associated psychopathologies. The literature shows that adolescents with high scores in IGD also have negative consequences at the psychosocial level: fewer recreational activities, fewer social activities and contacts, and diminished academic performance [86,87]. These abnormalities in “real-world” social support can affect people with different personality profiles in different ways. Generally, each online video game has an associated players’ community. This may lead players to find people online with similar interests and, thus, expand or replace their “real-life” social network. As these online relationships spend more and more time, “real-world” social relations will tend to deteriorate or disappear and this lack of “real-life” social support can lead some players to develop symptomatology. But in other cases, establishing this type of online relationships can help alleviate the psychological distress of some players, helping the person to establish social relationships through the Internet and build their lives around it. Some authors provide evidence that personality characteristics (e.g., extraversion, introversion) affect the choice of online or offline options for relationships [88].

Finally, age could be another key factor influencing comorbid psychopathology. In the present review, the strongest associations were found in the adult population. Results focusing on other behavioral addictions (i.e., Gambling Disorder), shows that younger adults, as opposed to older patients, only experience the symptoms of the addiction as psychological discomfort [89], without another comorbid psychopathology. One possible explanation is that older gamblers have experienced the negative consequences of the disorder for a longer period, and this has led them to develop comorbid psychopathology. It is also possible that the psychological symptoms associated with IGD require a longer time period to appear in certain subjects. Another hypothesis is that, first, children and adolescents tend to underestimate the long-term negative consequences of risky or prejudicial behaviors; and second, compared with adults, when making decisions adolescents tend to give more weight to short-term rewards compared with attendant risks [90]. Future research should analyze the differences in the perception of the negative consequences caused by IGD among adults and adolescents.

In relation to gender differences, similar to IA results all the reviewed studies reported higher video-game use among males, and most of the articles found a higher prevalence of IGD in males. Other authors have found that female respondents report less frequent play and less orientation to game genres featuring competition and three-dimensional rotation [91,92]. These characteristics in women players may be a protective factor against IGD. Regarding the amount of time spent playing, although contradictory results have been found regarding the relationship between this factor and IGD [31,42], some authors suggest that its control could be a protective factor in its appearance [93]. With respect to the type of video game chosen, it is likely that both the competitive factor and the immersive factor (in this case favored by a three-dimensional environment) of the online games, characteristics that women do not usually choose, may influence the development of IGD [94,95,96].

In order to clarify these points, future studies should focus on an analysis of the relationships between the personality of the affected people, the video-game preferences (e.g., massively multiplayer online role-playing game or MMORPG, multiplayer online battle arena or MOBA, first-person shooter gamers), the perception of the negative consequences generated by the problematic use, and the associated psychopathology.

The geographical distribution of the research in IGD seems to be more homogeneous than in IA; 50% of the included studies were developed in Europe and 50% were conducted in the rest of the world (29% in Asia, 30% in Australia, and 8% in North America). The prevalence of the problem and its correlation with psychopathology has been reported in all countries; therefore, it seems that it is a global problem and independent of cultural variation.

In contrast to IA, where there is a lack of common diagnostic criteria [14], in the case of IGD there are several questionnaires available based on the proposed diagnostic criteria for the disorder in the DSM-5. Despite this inclusion, the debate about the adequacy of these criteria and the emphasis upon online gaming rather than “general” gaming addiction is still active [97,98]. Therefore, although there is no gold standard questionnaire for IGD, the authors have a diagnostic base in which to frame their research. In the present review, of the 15 included articles published after the appearance of the DSM-5, only eight authors used these criteria or questionnaires based on them. The rest of the published research is based on measures for IA problems or questionnaires adapted from Gambling Disorder and general addiction criteria. This variability in evaluation methods, and basing the division of the comparison groups (IGD problems vs. no IGD problems) exclusively in the results of auto-administered data, could in part explain the variability found between IGD and comorbid psychopathology.

A consensus on the evaluation method of the problem is critical; in addition, studies focused on clinical populations with a diagnosis confirmed by professionals are needed. The data based on self-reports may not be accurate and may be limited in how they diagnose people [99]; therefore, in future research it would be helpful to complement the results of self-report questionnaires with clinical interviews (at least for the positive cases).

## 5. Limitations

The results of this review should be interpreted with several limitations in mind. First (as noted), some of the studies were published before the inclusion of IGD as a diagnostic category in the DSM-5. Thus, inconsistencies in clinical definitions and evaluations should be expected. Second, restrictions applied to the language of the articles, and heterogeneity in the nomenclature surrounding IGD across the different studies, suggests a potential risk that a relevant article was missed. However, articles written in other languages (with abstracts in English) were included in the review process; furthermore, a search in the citations of the selected literature was carried out. Third, reviewing only the first 30 pages of results in Google Scholar may have produced some bias; however, this method has been shown to be commonly used [100] and seems not to influence the results of the reviews. In addition, searches in other search engines and citations of included articles may have reduced that risk.

## 6. Conclusions

The present review included 24 studies analyzing the association between IGD and psychopathology. Compared with IA (which showed strong correlations only with depression), IGD showed strong correlations with anxiety, depression, ADHD or hyperactivity symptoms, social phobia/anxiety, and obsessive-compulsive symptoms. The lack of longitudinal studies and the contradictory results obtained makes it difficult to detect the directionality of these associations and shows the existing complexity of the relationship between IGD and psychopathology. In addition, due to a possible publication bias, the results should be interpreted with caution.

For future research, it would be helpful to investigate the relationships between personality styles, type of video-game problem, negative consequences, and associated psychopathology. It is also necessary to reach a consensus on the diagnostic criteria of IGD and on psychometric instruments used to research the subject. Studies centered in the clinical population, with diagnostic interviews that confirm the presence of the disorder, are critically needed.

## Figures and Tables

**Table 1:** Studies examining the relationship between Internet Gaming Disorder (IGD) and comorbid psychopathology, including effect sizes.

Source	Study Type	*N*	Population Age ^a^	Sex	Country	IGD Measures	Psychopathology Measures	Psychopathology Outcome	Association	Effect Size	95% CI of d
Baer et al., 2011 [24]	cross-sectional	102	adolescents 13.7 ± 1.9	M/F	Canada	Computer/Gaming-station Addiction Scale (CGAS)	Strengths and Difficulties Questionnaire	Emotional problems	full	R^2^ = 0.29	–
								Hyperactivity	full	R^2^ = 0.18	–
Cole & Hooley, 2013 [25]	cross-sectional	163	general population 27.3 ± 9.1	M/F	USA	Generalized Problematic Internet Use Scale (GPIUS)	State-Trait Anxiety Inventory (STAI)	Anxiety state	full	d = 0.26	−0.05–0.57
								Anxiety trait	full	d = 1.07	0.74–1.40
							Social Phobia Scale	Social phobia	full	d = 1.17	0.83–1.50
Jiménez-Murcia et al., 2014 [26]	cross-sectional	193	adults with GD 42.4 ± 13.4	M/F	Spain	Video-game Dependency Test (VDT)	Symptom Checklist 90-revision	Somatization	full	d = 0.57	0.16–0.983
								Obsessive-Compulsive	full	d = 0.84	0.424–1.257
								Interpersonal Sensitivity	full	d = 0.76	0.341–1.169
								Depression	full	d = 0.58	0.17–0.991
								Anxiety	full	d = 0.64	0.216–1.064
								Hostility	full	d = 0.68	0.255–1.106
								Phobic-Anxiety	full	d = 0.55	0.127–0.973
								Paranoid Ideation	full	d = 0.83	0.402–1.259
								Psychoticism	full	d = 0.56	0.137–0.983
Kim et al., 2016 [27]	cross-sectional	3041	adults 20–49	M/F	South Korea	IGD diagnostic criteria in DSM-5	Brief Symptom Inventory (BSI)	Somatization	full	d = 1.59	1.481–1.703
								Obsessive-Compulsive	full	d = 1.67	1.557–1.78
								Interpersonal Sensitivity	full	d = 1.61	1.499–1.721
								Depression	full	d = 1.75	1.642–1.867
								Anxiety	full	d = 1.75	1.642–1.866
								Hostility	full	d = 1.72	1.61–1.834
								Phobic-Anxiety	full	d = 1.82	1.705–1.928
								Paranoid Ideation	full	d = 1.74	1.623–1.847
								Psychoticism	full	d = 1.76	1.646–1.87
King et al., 2013 [28]	cross-sectional	1287	adolescents 12–18	M/F	Australia	Pathological Technology Use (PTU)	Revised Children’s Anxiety and Depression Scale	Depression	none	–	
								Obsessive-Compulsive Disorder (OCD)	none	–	
								Anxiety	none	–	
King & Delfabbro, 2016 [29]	cross-sectional	824	adolescents 14.1 ± 1.5	M/F	Australia	IGD Diagnostic criteria in DSM-5	Depression Anxiety Stress Scales, 21-item version	Depression	full *	d = 0.62	0.087–1.155
								Anxiety	full *	d = 0.50	−0.035–1.025
Laconi et al., 2017 [30]	cross-sectional	418	adults 21.9 ± 3	M/F	France	Internet Gaming Disorder Test-10 (IGDT-10)	Center for Epidemiologic Studies, Depression Scale-10	Depression	full	d = 2.687	1.969–3.405
Männikkö et al., 2015 [31]	cross-sectional	293	general population 18.7 ± 3.4	M/F	Finland	Gaming Addiction Scale (GAS)	School Health Promotion (SHP)	Depression	full	R^2^ = 0.17	-
								Anxiety	full	R^2^ = 0.11	-
Mentzoni, et al., 2011 [32]	cross-sectional	816	general population 15–40	M/F	Norway	Gaming Addiction Scale for Adolescents (GASA)	Hospital Anxiety and Depression Scale (HADS)	Depression	full	n/a	-
								Anxiety	full	n/a	-
Müller et al., 2015 [33]	cross-sectional	12,938	adolescents 15.8 ± 0.7	M/F	Germany	Assessment of Internet and Computer Game Addiction (AICGA)	Youth Self-Report	Anxious-Depression	full	d = 0.34	0.183–0.496
								Withdrawn-Depression	full	d = 0.35	0.347–0.507
Na et al., 2017 [34]	cross-sectional	1819	adults 20–49	M/F	South Korea	IGD diagnostic criteria in DSM-5	Symptom Checklist 90-revision	Depression	full	n/a	-
								Anxiety	full	n/a	-
Starcevic et al., 2011 [35]	cross-sectional	1945	general population over 14	M/F	Australia	Video-Game Use Questionnaire (VGUQ)	Symptom Checklist 90	Somatization	partial>	d = 1.02	0.854–1.187
								Obsessive-Compulsive	partial	d = 1.365	1.196–1.534
								Interpersonal Sensitivity	partial	d = 1.228	1.059–1.396
								Depression	partial	d = 1.264	1.096–1.433
								Anxiety	partial>	d = 1.149	0.981–1.317
								Hostility	partial>	d = 1.276	1.108–1.445
								Phobic-Anxiety	partial>	d = 1.131	0.964–1.299
								Paranoid Ideation	partial>	d = 1.203	1.035–1.371
								Psychoticism	partial>	d = 1.368	1.199–1.537
Stetina et al., 2011 [36]	cross-sectional	468	general population 11–67	M/F	Austria	Problematic Internet use scale (ISS-20)	Questionnaire for depression diagnostics (FDD for DSM-IV)	Depression	none	-	-
Strittmatter et al., 2015 [37]	cross-sectional	9758	adolescents 15.0 ± 1.3	M/F	Germany	Young Diagnostic Questionnaire (YDQ)	Beck Depression Inventory II	Depression	full	d = 0.58	0.449–0.702
							Strengths and Difficulties Questionnaire (SDQ)	Hyperactivity	full	d = 0.53	0.399–0.652
Vadlin et al., 2016 [38]	cross-sectional	*N1* (1868) *N2* (242)	adolescents 12–18	M/F	Sweden	Gaming Addiction Identification (GAIT)	Depression Self-Rating Scale (DSRS-A)	Depression	full	OR 2.47 (1.44–4.25)	-
							Spence Children‘s Anxiety Scale (SCAS)	Anxiety	full	OR 2.06 (1.27–3.33)	-
							Adult ADHD Self-Report Scale (ASRS-A)	Attention Deficit Hyperactivity Disorder (ADHD)	full	OR 2.43 (1.44–4.11)	-
							Psychotic-like experiences (PLEs)	Psychoticism	none	-	-
Wang et al., 2018 [39]	cross-sectional	7200	general population 14–39	M/F	South Korea	IGD diagnostic criteria in DSM-5	Patient Health Questionnaire9 (PHQ9)	Depression	full	n/a	-
							Generalized Anxiety Disorder Scale (GAD-7)	Anxiety	n/a	-	-
Wartberg et al., 2017 [40]	cross-sectional	1095	adolescents 13.0 ± 0.82	M/F	Germany	Internet Gaming Disorder Scale (IGDS)	Reynolds Adolescent Adjustment Screening Inventory	Depression and anxiety	full	OR 1.09 (1.02–1.17)	-
								Hyperactivity	full	OR 1.27 (1.16–1.39)	-
Wei et al., 2012 [41]	cross-sectional	722	general population 21.8 ± 4.9	M/F	Taiwan	Chen’s Internet Addiction Scale (CIAS)	Depression and Somatic Symptoms Scale (DSSS)	Depression	full	R^2^ = 0.298	-
							Social Phobia Inventory (SPIN)	Social phobia	full	n/a	-
Panagiotidi, 2017 [42]	cross-sectional	205	adults 27.4 ± 10	M/F	United Kingdom	Problem Video-Game Playing Test (PVGT)	ADHD Self-Report Scale (ASRS)	ADHD	full	R^2^ = 0.22	-
Gentile et al., 2011 [43]	Longitudinal	3034	children, adolescents 11.2 ± 2.06	M/F	Singapore	Pathological Technology Use (PTU)	Asian Adolescent Depression Scale (AADS)	Depression	full	R^2^ = 0.49	-
							Child Anxiety-Related Emotional Disorders (SCARED)	Anxiety	full	R^2^ = 0.29	-
							Adult ADHD Self-Report Scale (ASRS-A)	ADHD	none	-	-
							Social Phobia Inventory (SPIN)	Social phobia	full	R^2^ = 0.20	-
Van Rooij et al., 2011 [44]	Longitudinal	T1 (1572) T2 (1476)	children 13–16	M/F	Deutschland	Compulsive Internet Use Scale (CIUS)	Depressive Mood List	T1: Depression T2: Depression	none full #	n/a	-
							Revised Social Anxiety Scale for Children	T1: Social anxiety T2: Social anxiety	none none	-	-
Hyun et al., 2015 [45]	case-control	308	general population 21.0 ± 5.9	M/F	South Korea	Young Internet Addiction Scale (YIAS)	Beck Depressive Inventory (BDI)	Depression	full	d = 1.09	0.88–1.305
							Beck Anxiety Scale (BAI)	Anxiety	full	d = 0.64	0.437–0.845
							Dupaul’s ADHD scale (K-ARS)	ADHD	full	d = 1.05	0.838–1.262
Yen et al., 2016 [46]	case-control	174	adults 23.29 ± 2.34 23.38 ± 2.40	M/F	Taiwan	Semi-structured interview with the DSM-5 IGD criteria	ADHD DSM-IV-TR criteria diagnosis for adult and childhood	ADHD	full	OR 13.51 (4.49–40.64)	-
Brunborg et al., 2014 [47]	cohort	1928	adolescents 13–17	M/F	Norway	Game Addiction Scale for Adolescents (GASA)	Hopkins Symptom Checklist	Depression	T1: full other time: none	R^2^ = 0.25	- -

^a^ Age is presented in years as a range or mean with standard deviation (SD). M/F = both males and females analyzed together. * Low severity symptoms. n/a Non-enough data provided to calculate the effect size or not applicable. # When non-addicted heavy gamers and addicted heavy gamers compared. > A difference was found between IGD subjects and non IGD subjects but the psychopathology scores on both groups were not clinical.

**Table 2:** Number of observed associations identified between IGD and psychopathology stratified by effect size for the four main outcomes.

Effect Size	Depression	Anxiety	ADHD/Hyper-Activity	Social Phobia/Anxiety
Small ^a^	2	2	2	0
Moderate ^b^	3	5	1	0
Large ^c^	8	2	4	2
None	2	1	1	1
Total	15	10	8	3

^a^ d = 0.2, R^2^ = 0.01, OR = 1.45. ^b^ d = 0.5, R^2^ = 0.06, OR = 2.50. ^c^ d = 0.8, R^2^ = 0.14, OR = 4.25.
